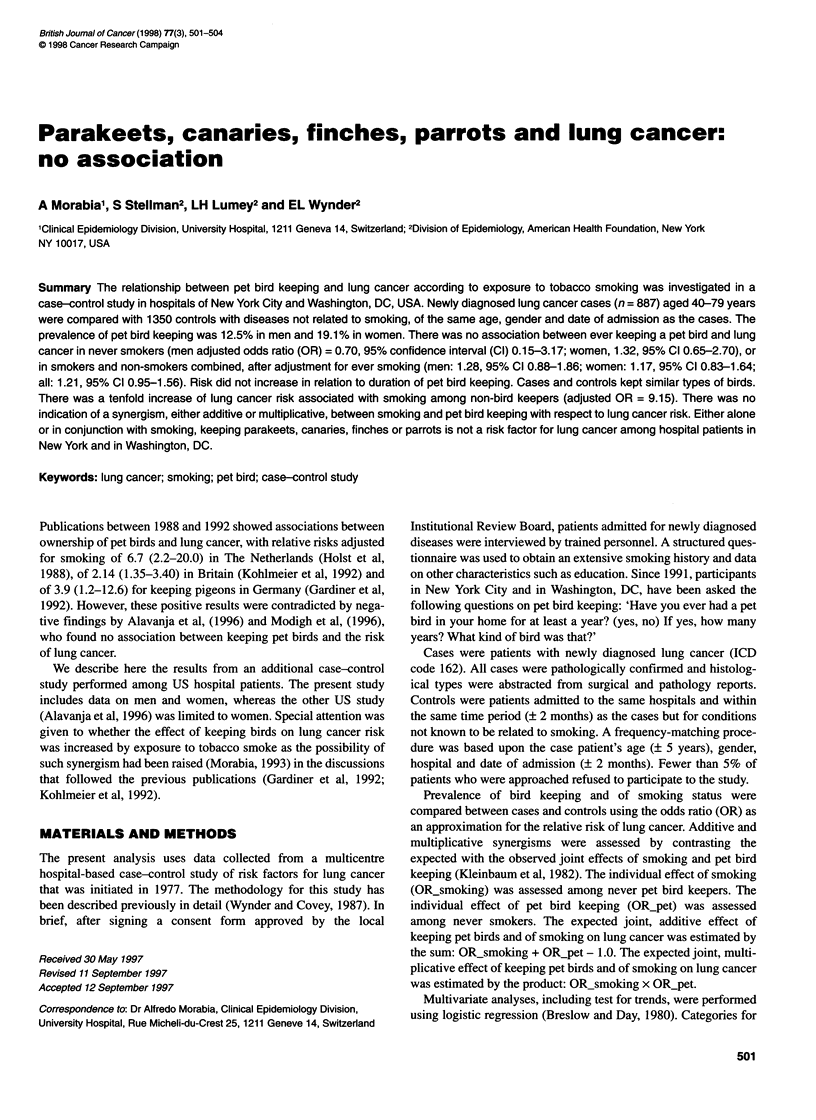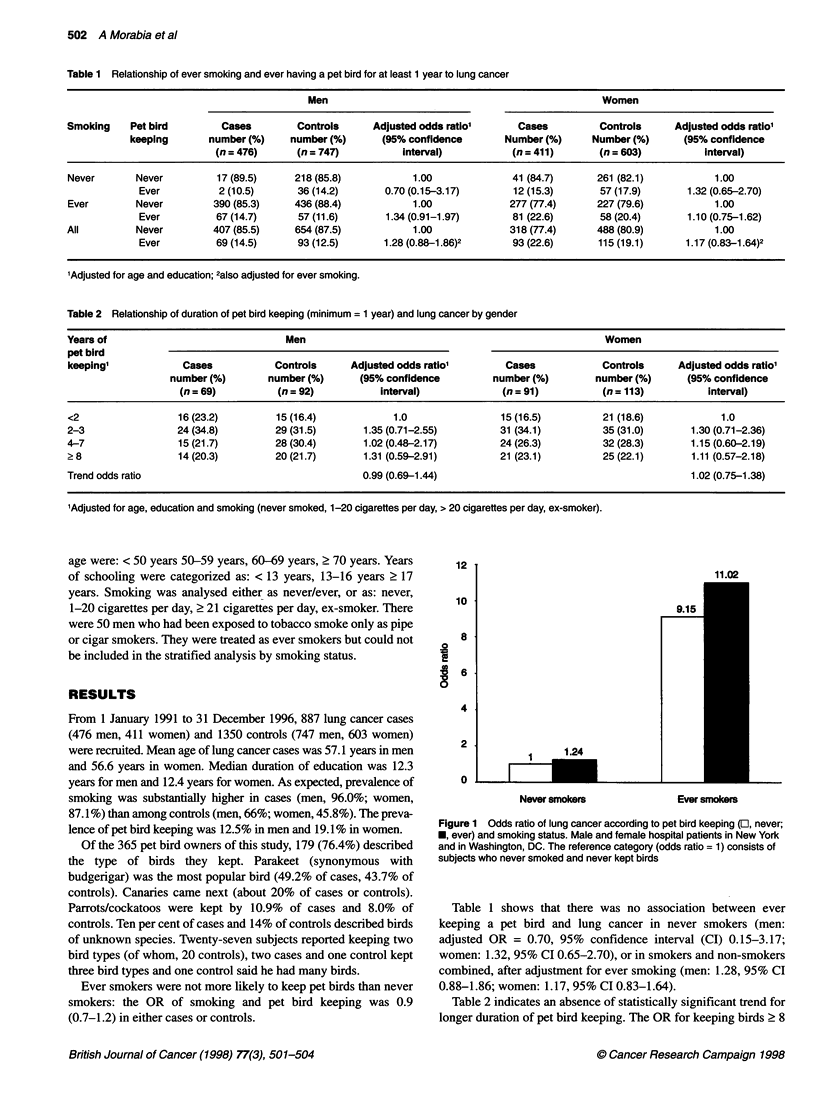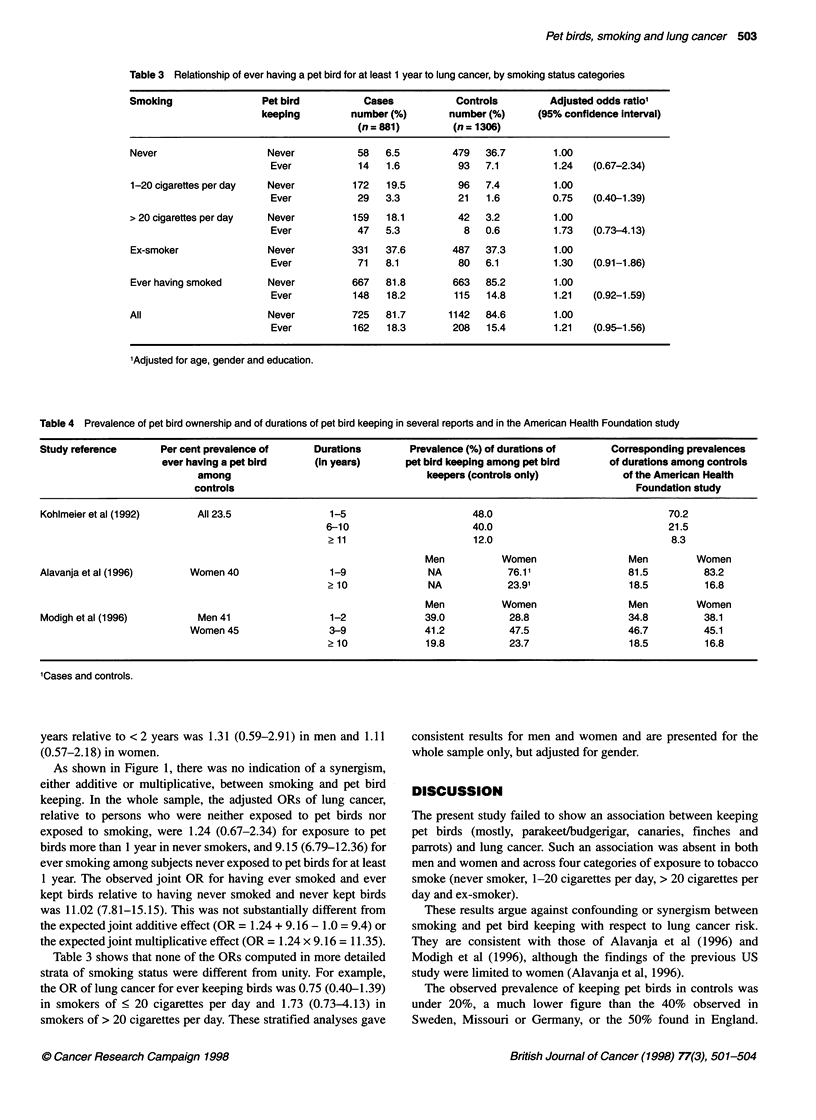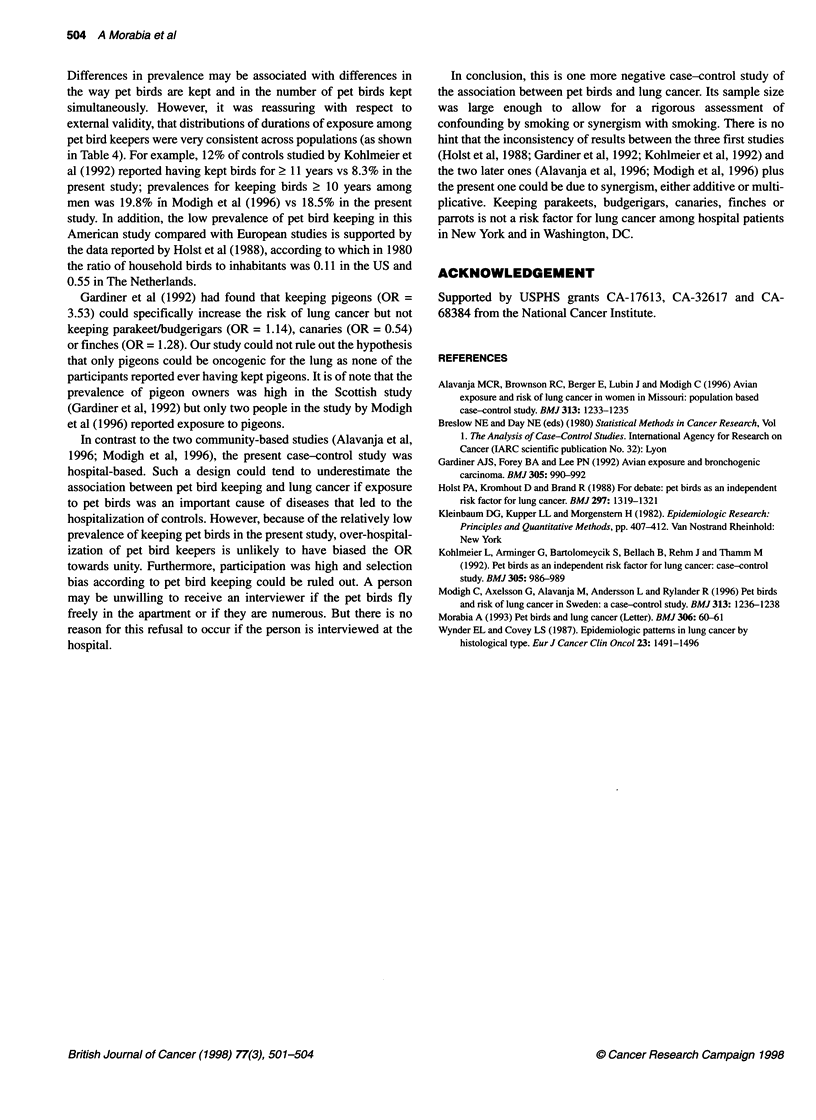# Parakeets, canaries, finches, parrots and lung cancer: no association.

**DOI:** 10.1038/bjc.1998.80

**Published:** 1998

**Authors:** A. Morabia, S. Stellman, L. H. Lumey, E. L. Wynder

**Affiliations:** Clinical Epidemiology Division, University Hospital, Geneva, Switzerland.

## Abstract

The relationship between pet bird keeping and lung cancer according to exposure to tobacco smoking was investigated in a case-control study in hospitals of New York City and Washington, DC, USA. Newly diagnosed lung cancer cases (n = 887) aged 40-79 years were compared with 1350 controls with diseases not related to smoking, of the same age, gender and date of admission as the cases. The prevalence of pet bird keeping was 12.5% in men and 19.1% in women. There was no association between ever keeping a pet bird and lung cancer in never smokers (men adjusted odds ratio (OR) = 0.70, 95% confidence interval (CI) 0.15-3.17; women, 1.32, 95% CI 0.65-2.70), or in smokers and non-smokers combined, after adjustment for ever smoking (men: 1.28, 95% CI 0.88-1.86; women: 1.17, 95% CI 0.83-1.64; all: 1.21, 95% CI 0.95-1.56). Risk did not increase in relation to duration of pet bird keeping. Cases and controls kept similar types of birds. There was a tenfold increase of lung cancer risk associated with smoking among non-bird keepers (adjusted OR = 9.15). There was no indication of a synergism, either additive or multiplicative, between smoking and pet bird keeping with respect to lung cancer risk. Either alone or in conjunction with smoking, keeping parakeets, canaries, finches or parrots is not a risk factor for lung cancer among hospital patients in New York and in Washington, DC.


British Journal of Cancer (1998) 77(3), 501–504
© 1998 Cancer Research Campaign

### Parakeets, canaries, finches, parrots and lung cancer: no association

**A Morabia[1], S Stellman[2], LH Lumey[2] and EL Wynder[2]**

[1]Clinical Epidemiology Division, University Hospital, 1211 Geneva 14, Switzerland; [2]Division of Epidemiology, American Health Foundation, New York NY 10017, USA

**Summary** The relationship between pet bird keeping and lung cancer according to exposure to tobacco smoking was investigated in a case–control study in hospitals of New York City and Washington, DC, USA. Newly diagnosed lung cancer cases ($n$ = 887) aged 40–79 years were compared with 1350 controls with diseases not related to smoking, of the same age, gender and date of admission as the cases. The prevalence of pet bird keeping was 12.5% in men and 19.1% in women. There was no association between ever keeping a pet bird and lung cancer in never smokers (men adjusted odds ratio (OR) = 0.70, 95% confidence interval (CI) 0.15–3.17; women, 1.32, 95% CI 0.65–2.70), or in smokers and non-smokers combined, after adjustment for ever smoking (men: 1.28, 95% CI 0.88–1.86; women: 1.17, 95% CI 0.83–1.64; all: 1.21, 95% CI 0.95–1.56). Risk did not increase in relation to duration of pet bird keeping. Cases and controls kept similar types of birds. There was a tenfold increase of lung cancer risk associated with smoking among non-bird keepers (adjusted OR = 9.15). There was no indication of a synergism, either additive or multiplicative, between smoking and pet bird keeping with respect to lung cancer risk. Either alone or in conjunction with smoking, keeping parakeets, canaries, finches or parrots is not a risk factor for lung cancer among hospital patients in New York and in Washington, DC.

**Keywords:** lung cancer; smoking; pet bird; case–control study

Publications between 1988 and 1992 showed associations between ownership of pet birds and lung cancer, with relative risks adjusted for smoking of 6.7 (2.2–20.0) in The Netherlands (Holst et al, 1988), of 2.14 (1.35–3.40) in Britain (Kohlmeier et al, 1992) and of 3.9 (1.2–12.6) for keeping pigeons in Germany (Gardiner et al, 1992). However, these positive results were contradicted by negative findings by Alavanja et al, (1996) and Modigh et al, (1996), who found no association between keeping pet birds and the risk of lung cancer.

We describe here the results from an additional case–control study performed among US hospital patients. The present study includes data on men and women, whereas the other US study (Alavanja et al, 1996) was limited to women. Special attention was given to whether the effect of keeping birds on lung cancer risk was increased by exposure to tobacco smoke as the possibility of such synergism had been raised (Morabia, 1993) in the discussions that followed the previous publications (Gardiner et al, 1992; Kohlmeier et al, 1992).

### MATERIALS AND METHODS

The present analysis uses data collected from a multicentre hospital-based case–control study of risk factors for lung cancer that was initiated in 1977. The methodology for this study has been described previously in detail (Wynder and Covey, 1987). In brief, after signing a consent form approved by the local Institutional Review Board, patients admitted for newly diagnosed diseases were interviewed by trained personnel. A structured questionnaire was used to obtain an extensive smoking history and data on other characteristics such as education. Since 1991, participants in New York City and in Washington, DC, have been asked the following questions on pet bird keeping: ‘Have you ever had a pet bird in your home for at least a year? (yes, no) If yes, how many years? What kind of bird was that?’

Cases were patients with newly diagnosed lung cancer (ICD code 162). All cases were pathologically confirmed and histological types were abstracted from surgical and pathology reports. Controls were patients admitted to the same hospitals and within the same time period (± 2 months) as the cases but for conditions not known to be related to smoking. A frequency-matching procedure was based upon the case patient's age (± 5 years), gender, hospital and date of admission (± 2 months). Fewer than 5% of patients who were approached refused to participate to the study.

Prevalence of bird keeping and of smoking status were compared between cases and controls using the odds ratio (OR) as an approximation for the relative risk of lung cancer. Additive and multiplicative synergisms were assessed by contrasting the expected with the observed joint effects of smoking and pet bird keeping (Kleinbaum et al, 1982). The individual effect of smoking (OR_smoking) was assessed among never pet bird keepers. The individual effect of pet bird keeping (OR_pet) was assessed among never smokers. The expected joint, additive effect of keeping pet birds and of smoking on lung cancer was estimated by the sum: OR_smoking + OR_pet – 1.0. The expected joint, multiplicative effect of keeping pet birds and of smoking on lung cancer was estimated by the product: OR_smoking × OR_pet.

Multivariate analyses, including test for trends, were performed using logistic regression (Breslow and Day, 1980). Categories for

*Received 30 May 1997*
*Revised 11 September 1997*
*Accepted 12 September 1997*

*Correspondence to*: Dr Alfredo Morabia, Clinical Epidemiology Division, University Hospital, Rue Micheli-du-Crest 25, 1211 Geneve 14, Switzerland

**Table 1** Relationship of ever smoking and ever having a pet bird for at least 1 year to lung cancer

| Smoking | Pet bird keeping | Men: Cases number (%) (*n* = 476) | Men: Controls number (%) (*n* = 747) | Men: Adjusted odds ratio[1] (95% confidence interval) | Women: Cases Number (%) (*n* = 411) | Women: Controls Number (%) (*n* = 603) | Women: Adjusted odds ratio[1] (95% confidence interval) |
|---|---|---|---|---|---|---|---|
| Never | Never | 17 (89.5) | 218 (85.8) | 1.00 | 41 (84.7) | 261 (82.1) | 1.00 |
| | Ever | 2 (10.5) | 36 (14.2) | 0.70 (0.15–3.17) | 12 (15.3) | 57 (17.9) | 1.32 (0.65–2.70) |
| Ever | Never | 390 (85.3) | 436 (88.4) | 1.00 | 277 (77.4) | 227 (79.6) | 1.00 |
| | Ever | 67 (14.7) | 57 (11.6) | 1.34 (0.91–1.97) | 81 (22.6) | 58 (20.4) | 1.10 (0.75–1.62) |
| All | Never | 407 (85.5) | 654 (87.5) | 1.00 | 318 (77.4) | 488 (80.9) | 1.00 |
| | Ever | 69 (14.5) | 93 (12.5) | 1.28 (0.88–1.86)[2] | 93 (22.6) | 115 (19.1) | 1.17 (0.83–1.64)[2] |

[1]Adjusted for age and education; [2]also adjusted for ever smoking.

**Table 2** Relationship of duration of pet bird keeping (minimum = 1 year) and lung cancer by gender

| Years of pet bird keeping[1] | Men: Cases number (%) (*n* = 69) | Men: Controls number (%) (*n* = 92) | Men: Adjusted odds ratio[1] (95% confidence interval) | Women: Cases number (%) (*n* = 91) | Women: Controls number (%) (*n* = 113) | Women: Adjusted odds ratio[1] (95% confidence interval) |
|---|---|---|---|---|---|---|
| <2 | 16 (23.2) | 15 (16.4) | 1.0 | 15 (16.5) | 21 (18.6) | 1.0 |
| 2–3 | 24 (34.8) | 29 (31.5) | 1.35 (0.71–2.55) | 31 (34.1) | 35 (31.0) | 1.30 (0.71–2.36) |
| 4–7 | 15 (21.7) | 28 (30.4) | 1.02 (0.48–2.17) | 24 (26.3) | 32 (28.3) | 1.15 (0.60–2.19) |
| ≥ 8 | 14 (20.3) | 20 (21.7) | 1.31 (0.59–2.91) | 21 (23.1) | 25 (22.1) | 1.11 (0.57–2.18) |
| Trend odds ratio | | | 0.99 (0.69–1.44) | | | 1.02 (0.75–1.38) |

[1]Adjusted for age, education and smoking (never smoked, 1–20 cigarettes per day, > 20 cigarettes per day, ex-smoker).

age were: < 50 years 50–59 years, 60–69 years, ≥ 70 years. Years of schooling were categorized as: < 13 years, 13–16 years ≥ 17 years. Smoking was analysed either as never/ever, or as: never, 1–20 cigarettes per day, ≥ 21 cigarettes per day, ex-smoker. There were 50 men who had been exposed to tobacco smoke only as pipe or cigar smokers. They were treated as ever smokers but could not be included in the stratified analysis by smoking status.

### RESULTS

From 1 January 1991 to 31 December 1996, 887 lung cancer cases (476 men, 411 women) and 1350 controls (747 men, 603 women) were recruited. Mean age of lung cancer cases was 57.1 years in men and 56.6 years in women. Median duration of education was 12.3 years for men and 12.4 years for women. As expected, prevalence of smoking was substantially higher in cases (men, 96.0%; women, 87.1%) than among controls (men, 66%; women, 45.8%). The prevalence of pet bird keeping was 12.5% in men and 19.1% in women.

Of the 365 pet bird owners of this study, 179 (76.4%) described the type of birds they kept. Parakeet (synonymous with budgerigar) was the most popular bird (49.2% of cases, 43.7% of controls). Canaries came next (about 20% of cases or controls). Parrots/cockatoos were kept by 10.9% of cases and 8.0% of controls. Ten per cent of cases and 14% of controls described birds of unknown species. Twenty-seven subjects reported keeping two bird types (of whom, 20 controls), two cases and one control kept three bird types and one control said he had many birds.

Ever smokers were not more likely to keep pet birds than never smokers: the OR of smoking and pet bird keeping was 0.9 (0.7–1.2) in either cases or controls.

**Figure 1** Odds ratio of lung cancer according to pet bird keeping (□, never; ■, ever) and smoking status. Male and female hospital patients in New York and in Washington, DC. The reference category (odds ratio = 1) consists of subjects who never smoked and never kept birds

Table 1 shows that there was no association between ever keeping a pet bird and lung cancer in never smokers (men: adjusted OR = 0.70, 95% confidence interval (CI) 0.15–3.17; women: 1.32, 95% CI 0.65–2.70), or in smokers and non-smokers combined, after adjustment for ever smoking (men: 1.28, 95% CI 0.88–1.86; women: 1.17, 95% CI 0.83–1.64).

Table 2 indicates an absence of statistically significant trend for longer duration of pet bird keeping. The OR for keeping birds ≥ 8

© Cancer Research Campaign 1998

**Table 3** Relationship of ever having a pet bird for at least 1 year to lung cancer, by smoking status categories

| Smoking | Pet bird keeping | Cases number (%) (*n* = 881) | | Controls number (%) (*n* = 1306) | | Adjusted odds ratio[1] (95% confidence interval) | |
|---|---|---|---|---|---|---|---|
| Never | Never | 58 | 6.5 | 479 | 36.7 | 1.00 | |
| | Ever | 14 | 1.6 | 93 | 7.1 | 1.24 | (0.67–2.34) |
| 1–20 cigarettes per day | Never | 172 | 19.5 | 96 | 7.4 | 1.00 | |
| | Ever | 29 | 3.3 | 21 | 1.6 | 0.75 | (0.40–1.39) |
| > 20 cigarettes per day | Never | 159 | 18.1 | 42 | 3.2 | 1.00 | |
| | Ever | 47 | 5.3 | 8 | 0.6 | 1.73 | (0.73–4.13) |
| Ex-smoker | Never | 331 | 37.6 | 487 | 37.3 | 1.00 | |
| | Ever | 71 | 8.1 | 80 | 6.1 | 1.30 | (0.91–1.86) |
| Ever having smoked | Never | 667 | 81.8 | 663 | 85.2 | 1.00 | |
| | Ever | 148 | 18.2 | 115 | 14.8 | 1.21 | (0.92–1.59) |
| All | Never | 725 | 81.7 | 1142 | 84.6 | 1.00 | |
| | Ever | 162 | 18.3 | 208 | 15.4 | 1.21 | (0.95–1.56) |

[1]Adjusted for age, gender and education.

**Table 4** Prevalence of pet bird ownership and of durations of pet bird keeping in several reports and in the American Health Foundation study

| Study reference | Per cent prevalence of ever having a pet bird among controls | Durations (in years) | Prevalence (%) of durations of pet bird keeping among pet bird keepers (controls only) | | Corresponding prevalences of durations among controls of the American Health Foundation study | |
|---|---|---|---|---|---|---|
| Kohlmeier et al (1992) | All 23.5 | 1–5 | 48.0 | | 70.2 | |
| | | 6–10 | 40.0 | | 21.5 | |
| | | ≥ 11 | 12.0 | | 8.3 | |
| | | | Men | Women | Men | Women |
| Alavanja et al (1996) | Women 40 | 1–9 | NA | 76.1[1] | 81.5 | 83.2 |
| | | ≥ 10 | NA | 23.9[1] | 18.5 | 16.8 |
| | | | Men | Women | Men | Women |
| Modigh et al (1996) | Men 41 | 1–2 | 39.0 | 28.8 | 34.8 | 38.1 |
| | Women 45 | 3–9 | 41.2 | 47.5 | 46.7 | 45.1 |
| | | ≥ 10 | 19.8 | 23.7 | 18.5 | 16.8 |

[1]Cases and controls.

years relative to < 2 years was 1.31 (0.59–2.91) in men and 1.11 (0.57–2.18) in women.

As shown in Figure 1, there was no indication of a synergism, either additive or multiplicative, between smoking and pet bird keeping. In the whole sample, the adjusted ORs of lung cancer, relative to persons who were neither exposed to pet birds nor exposed to smoking, were 1.24 (0.67–2.34) for exposure to pet birds more than 1 year in never smokers, and 9.15 (6.79–12.36) for ever smoking among subjects never exposed to pet birds for at least 1 year. The observed joint OR for having ever smoked and ever kept birds relative to having never smoked and never kept birds was 11.02 (7.81–15.15). This was not substantially different from the expected joint additive effect (OR = 1.24 + 9.16 – 1.0 = 9.4) or the expected joint multiplicative effect (OR = 1.24 × 9.16 = 11.35).

Table 3 shows that none of the ORs computed in more detailed strata of smoking status were different from unity. For example, the OR of lung cancer for ever keeping birds was 0.75 (0.40–1.39) in smokers of ≤ 20 cigarettes per day and 1.73 (0.73–4.13) in smokers of > 20 cigarettes per day. These stratified analyses gave consistent results for men and women and are presented for the whole sample only, but adjusted for gender.

### DISCUSSION

The present study failed to show an association between keeping pet birds (mostly, parakeet/budgerigar, canaries, finches and parrots) and lung cancer. Such an association was absent in both men and women and across four categories of exposure to tobacco smoke (never smoker, 1–20 cigarettes per day, > 20 cigarettes per day and ex-smoker).

These results argue against confounding or synergism between smoking and pet bird keeping with respect to lung cancer risk. They are consistent with those of Alavanja et al (1996) and Modigh et al (1996), although the findings of the previous US study were limited to women (Alavanja et al, 1996).

The observed prevalence of keeping pet birds in controls was under 20%, a much lower figure than the 40% observed in Sweden, Missouri or Germany, or the 50% found in England.

Differences in prevalence may be associated with differences in the way pet birds are kept and in the number of pet birds kept simultaneously. However, it was reassuring with respect to external validity, that distributions of durations of exposure among pet bird keepers were very consistent across populations (as shown in Table 4). For example, 12% of controls studied by Kohlmeier et al (1992) reported having kept birds for ≥ 11 years vs 8.3% in the present study; prevalences for keeping birds ≥ 10 years among men was 19.8% in Modigh et al (1996) vs 18.5% in the present study. In addition, the low prevalence of pet bird keeping in this American study compared with European studies is supported by the data reported by Holst et al (1988), according to which in 1980 the ratio of household birds to inhabitants was 0.11 in the US and 0.55 in The Netherlands.

Gardiner et al (1992) had found that keeping pigeons (OR = 3.53) could specifically increase the risk of lung cancer but not keeping parakeet/budgerigars (OR = 1.14), canaries (OR = 0.54) or finches (OR = 1.28). Our study could not rule out the hypothesis that only pigeons could be oncogenic for the lung as none of the participants reported ever having kept pigeons. It is of note that the prevalence of pigeon owners was high in the Scottish study (Gardiner et al, 1992) but only two people in the study by Modigh et al (1996) reported exposure to pigeons.

In contrast to the two community-based studies (Alavanja et al, 1996; Modigh et al, 1996), the present case–control study was hospital-based. Such a design could tend to underestimate the association between pet bird keeping and lung cancer if exposure to pet birds was an important cause of diseases that led to the hospitalization of controls. However, because of the relatively low prevalence of keeping pet birds in the present study, over-hospitalization of pet bird keepers is unlikely to have biased the OR towards unity. Furthermore, participation was high and selection bias according to pet bird keeping could be ruled out. A person may be unwilling to receive an interviewer if the pet birds fly freely in the apartment or if they are numerous. But there is no reason for this refusal to occur if the person is interviewed at the hospital.

In conclusion, this is one more negative case–control study of the association between pet birds and lung cancer. Its sample size was large enough to allow for a rigorous assessment of confounding by smoking or synergism with smoking. There is no hint that the inconsistency of results between the three first studies (Holst et al, 1988; Gardiner et al, 1992; Kohlmeier et al, 1992) and the two later ones (Alavanja et al, 1996; Modigh et al, 1996) plus the present one could be due to synergism, either additive or multiplicative. Keeping parakeets, budgerigars, canaries, finches or parrots is not a risk factor for lung cancer among hospital patients in New York and in Washington, DC.

### ACKNOWLEDGEMENT

Supported by USPHS grants CA-17613, CA-32617 and CA-68384 from the National Cancer Institute.

### REFERENCES

Alavanja MCR, Brownson RC, Berger E, Lubin J and Modigh C (1996) Avian exposure and risk of lung cancer in women in Missouri: population based case–control study. *BMJ* **313:** 1233–1235

Breslow NE and Day NE (eds) (1980) *Statistical Methods in Cancer Research*, Vol 1. *The Analysis of Case–Control Studies*. International Agency for Research on Cancer (IARC scientific publication No. 32): Lyon

Gardiner AJS, Forey BA and Lee PN (1992) Avian exposure and bronchogenic carcinoma. *BMJ* **305:** 990–992

Holst PA, Kromhout D and Brand R (1988) For debate: pet birds as an independent risk factor for lung cancer. *BMJ* **297:** 1319–1321

Kleinbaum DG, Kupper LL and Morgenstern H (1982). *Epidemiologic Research: Principles and Quantitative Methods*, pp. 407–412. Van Nostrand Rheinhold: New York

Kohlmeier L, Arminger G, Bartolomeycik S, Bellach B, Rehm J and Thamm M (1992). Pet birds as an independent risk factor for lung cancer: case–control study. *BMJ* **305:** 986–989

Modigh C, Axelsson G, Alavanja M, Andersson L and Rylander R (1996) Pet birds and risk of lung cancer in Sweden: a case–control study. *BMJ* **313:** 1236–1238

Morabia A (1993) Pet birds and lung cancer (Letter). *BMJ* **306:** 60–61

Wynder EL and Covey LS (1987). Epidemiologic patterns in lung cancer by histological type. *Eur J Cancer Clin Oncol* **23:** 1491–1496

© Cancer Research Campaign 1998